# Characterization of the time course of changes of the evoked electrical activity in a model of a chemically-induced neuronal plasticity

**DOI:** 10.1186/1756-0500-2-13

**Published:** 2009-01-27

**Authors:** Frédéric D Broccard, Silvia Pegoraro, Maria Elisabetta Ruaro, Claudio Altafini, Vincent Torre

**Affiliations:** 1 International School for Advanced Studies, Area Science Park SS 14 Km 163.5 Edificio Q1 34012- Basovizza, Trieste, Italy; 2 Glance Vision Technologies, Area Science Park SS 14 Km 163.5 Edificio Q 34012- Basovizza, Trieste, Italy; 3 IIT SISSA Unit Trieste, via Beirut 2-4, 34014 Trieste, Italy

## Abstract

**Background:**

Neuronal plasticity is initiated by transient elevations of neuronal networks activity leading to changes of synaptic properties and providing the basis for memory and learning [1]. An increase of electrical activity can be caused by electrical stimulation [2] or by pharmacological manipulations: elevation of extracellular K^+ ^[3], blockage of inhibitory pathways [4] or by an increase of second messengers intracellular concentrations [5]. Neuronal plasticity is mediated by several biochemical pathways leading to the modulation of synaptic strength, density of ionic channels and morphological changes of neuronal arborisation [6]. On a time scale of a few minutes, neuronal plasticity is mediated by local protein trafficking [7] while, in order to sustain modifications beyond 2–3 h, changes of gene expression are required [8].

**Findings:**

In the present manuscript we analysed the time course of changes of the evoked electrical activity during neuronal plasticity and we correlated it with a transcriptional analysis of the underlying changes of gene expression. Our investigation shows that treatment for 30 min. with the GABA_A _receptor antagonist gabazine (GabT) causes a potentiation of the evoked electrical activity occurring 2–4 hours after GabT and the concomitant up-regulation of 342 genes. Inhibition of the ERK1/2 pathway reduced but did not abolish the potentiation of the evoked response caused by GabT. In fact not all the genes analysed were blocked by ERK1/2 inhibitors.

**Conclusion:**

These results are in agreement with the notion that neuronal plasticity is mediated by several distinct pathways working in unison.

## Findings

**Figure 1:**
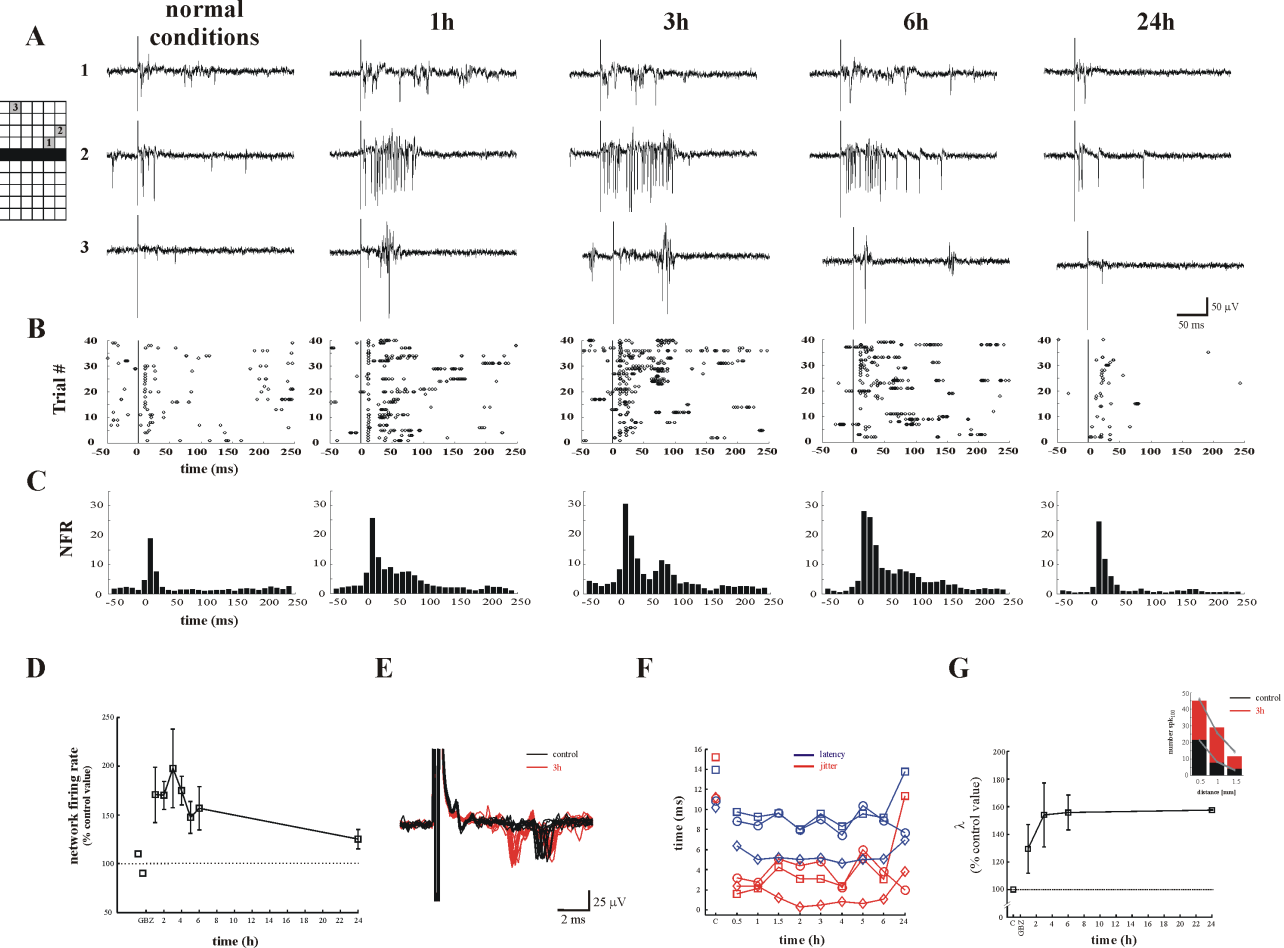
**Potentiation of the evoked response induced by GabT**. ***A***, Examples of the evoked activity's time course for a single trial for three representative electrodes located at 0.5, 1 and 2 mm distance (see *inset*) from the stimulating electrode respectively (black bar in *inset*). The number of evoked spikes increases following the GabT. Hippocampal cultures were stimulated with the lowest intensity (200–450 mV) evoking a response. 40 pulses (trials) with an inter-pulse interval 4s were used. Inset, graphical representation of the MEA grid. Each square represents an electrode. Distance between electrodes is 500 μm. Black bar corresponds to the stimulated electrodes and grey numbered squares to the electrodes whose activity is shown in ***(A)***. ***B***, Raster plots of the spikes evoked in one electrode analyzed at each time point. Each horizontal line represents the response to one trial recorded up to 250 ms after the stimulus onset. ***C***, Time course of the NFR. The total number of evoked spikes was counted in 10 ms bins, following electrical stimulation. The increase of evoked spikes is especially pronounced in the first 100 ms following stimulation. Data from a single experiment shown in ***(A-C)***. ***D***, Average time course of the NFR (n = 5 cultures). The NFR was normalized relative to control values. Bin size was 250 ms. ***E***, Ten overlapping spike traces in control conditions (black traces) and 3 h after gabazine washout (red traces) of an individual electrode showing the decrease of the latency of the first evoked spike. Artifacts have been truncated for clarity. ***F***, Time course of the latency (blue traces) and jitter (red traces) of the first spike for three neurons showing that latency and jitter decrease after GabT. Each symbol corresponds to a different neuron. ***G***, Time course of propagation constant (λ see Methods) showing the increase of the activity spread following GabT. Inset, the number of spikes in a 100 ms time window was counted and averaged, for electrodes located at 0.5, 1 and 2 mm from the stimulated bar of electrodes, and fitted with an exponential function *Ae*^-*d*/λ ^(grey line). Colours as in ***(E)***.

We have used dissociated neuronal cultures grown over MEA for 2–6 weeks to monitor the electrical activity from a population of neurons [9]. MEAs allow stable and long lasting recordings (hours to days) of extracellular signals from the entire population and permit to characterize and follow the properties of single spikes from identified neurons. In this way, it was possible to describe the global properties of the network, such as its overall electrical activity and to obtain a characterization of changes during neuronal plasticity of single identified spikes. This analysis could not be performed with hippocampal slices or organotypic cultures grown on MEAs or *in vivo*, because in these cases local field potentials (LFPs) are observed and a detailed investigation of neuronal plasticity at a single spike level is almost impossible. We increased synaptic efficacy and the overall electrical activity by treating hippocampal cultures for 30 min. with the GABA_A _receptor antagonist gabazine (GabT). After GabT, gabazine was washed out and the time course of evoked electrical activity was followed/studied. MEA's extracellular electrodes were used for recording and stimulation so to quantify changes of the evoked activity. Brief (200 μs) bipolar pulses were applied to a row of electrodes (black bar in the grid of Fig. 1A) and the propagation of evoked spikes throughout the network was recorded. In order to avoid saturation, the lowest voltage pulse evoking at least one spike was used, before, during and after GabT (Fig. 1). Its amplitude varied between 200 to 450 mV depending on the culture. After GabT, the number of evoked spikes increased at all times in almost all trials at the level of the single electrode (Fig. 1B) and in the entire network (Fig. 1C).

Changes of the evoked response were quantified by computing the total number of evoked spikes in a time window of 100 ms from the stimulus onset, referred to as the network firing rate of the evoked response NFR. NFR significantly increased between 1 and 6 h after GabT (Fig. 1C, D): the evoked response was maximally potentiated 3 h after GabT and started to decline after 6 h, returning close to the control level 24 h after GabT.

Neuronal plasticity induced by GabT not only modified synaptic efficacy but also several other network properties such as speed and reliability of the evoked spikes. Speed was evaluated by measuring the latency of the evoked response i.e. the delay from the stimulus of the evoked spike, while reliability was measured by the standard deviation of the latency (jitter). In some experiments it was possible to identify spikes produced by the same neuron (Fig. 1E) and therefore we could measure how its latency and jitter changed during neuronal plasticity. The latency in control conditions from stimulus onset varied between 6 and 9 ms, was reduced by 2–3 ms after GabT and its jitter similarly decreased (Fig. 1F). We also analyzed how spikes propagated in the network by measuring the space constant λ (total number of evoked spikes as a function of the distance from the stimulus) of the evoked activity. Collected data from 4 cultures showed that λ increased by about 25 % within 1 h after GabT and remained larger than the control values up to 24 h (Fig. 1G). These results show that increase of synaptic efficacy by exposure to gabazine alone in the absence of a concomitant strong or tetanic electrical stimulation, potentiated the electrical response propagating in the culture, inducing in this way a form of LTP, which we refer to as medium time LTP (M-LTP) because it was not identified as maximal after gabazine removal, developed 1 hour after GabT and lasted about 6 hours. Therefore, M-LTP is likely to be associated not only to local protein trafficking but also to changes of gene expression, occurring on a time scale of some hours. In order to understand the molecular events underlying M-LTP, we have analysed changes of gene expression induced by the same pharmacological treatment, i.e. a 30 min. exposure to gabazine with Affymetrix microarrays (RAT 230_2.0 Gene Chip) (Broccard et al., manuscript in preparation). We have identified 342 genes significantly up-regulated at the same times as when the evoked electrical activity was potentiated. Nevertheless, because gene profiles were obtained from the whole culture, we could not identify the type of neurons where up-regulated genes were expressed. Many of these genes are well known players in LTP such as *Bdnf *and its receptor TrkB [11], *Arc *[12], *Egr1 *[13] and *Homer1 *[14]. We hypothesized that the large majority of identified genes underlies induction and maintenance of LTP and that their activation orchestrates neuronal plasticity. In fact, a search in the PuBMed database indicates that 40% of the 284 annotated genes is, or could be, involved in changes of synaptic strength related to LTP. 43 genes have already been implicated in LTP, 25 genes have been classified as *Structural genes *for their structural role in cellular function and their up-regulation could underlie structural and morphological changes associated to LTP. Analogously, the 25 *Pre-synaptic *and the 24 *Post-synaptic *genes found in our screening could mediate changes of synaptic properties occurring during LTP.

**Figure 2:**
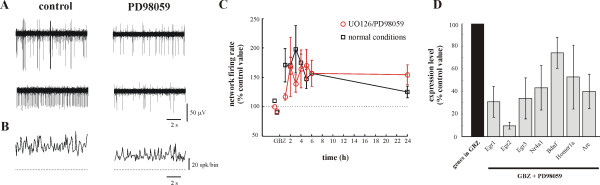
**Effect of inhibitors of the ERK1/2 pathway**. ***A***, Representative traces from two individual extracellular electrodes in control conditions (left) and in the presence of 50 μM PD98059 (right). ***B***, Corresponding network firing rate computed with a bin width of 25 ms, in normal conditions (left) and in the presence of 50 μM PD98059 (right) showing a depression of the spontaneous electrical activity by PD98059. Dotted line indicates zero spike. ***C***, The evoked activity is still potentiated when the ERK1/2 pathway was blocked before GabT (see Results). Dashed line corresponds to potentiation of the evoked activity without blocking the ERK1/2 pathway before GabT. Data for PD98059 (50 μM; n = 3) and U0126 (20 μM; n = 3) were pooled together as they affected network properties in a similar way. ***D***, Changes of gene expression occurring 1.5 h after GabT measured by Real-Time PCR for *Egr1*, *Egr2*, *Egr3*, *Nr4a1*, *Bdnf*, *Homer1a *and *Arc *in the presence of gabazine and PD98059 (gray bars) relative to normalized expression in the presence of gabazine alone (black bar).

ERK1/2 signalling plays an important role in several plasticity-related processes in the nervous system [15]. Therefore, we investigated the effect of the inhibition of ERK1/2 pathway by PD98059 and U0126 on the potentiation of the evoked response (Fig. 2). Application of these inhibitors to neuronal cultures decreased the spontaneous activity measured for all extracellular electrodes (Fig. 2A). The network firing rate was almost halved (Fig. 2B) in all tested cultures (n = 4), but periods of larger electrical activity could still be observed. In these cultures, inhibitors of the ERK1/2 were incubated for 45 minutes before GabT and changes of the evoked response were analyzed. In the presence of these inhibitors, GabT still potentiated the evoked response, although to a lesser extent (red trace in Fig. 2C), with a time course similar to that observed in the absence of these inhibitors (black trace in Fig. 2C). 3 h after GabT, the number of evoked spikes reached 198 ± 41 % in normal conditions, but increased only by 39 ± 15 % in the presence of PD98059 and U0126. Therefore, inhibition of the ERK1/2 pathway reduced but not abolished the potentiation of the evoked response caused by GabT.

We analyzed with real-time PCR the effect of the ERK1/2 inhibitors on some of the LTP-related genes, up-regulated in our microarray screening: *Egr1 *[13],*Egr2 *[16],*Egr3 *[17],*Nr4a1 *[18],*Bdnf *[11],*Homer1a *[14] and *Arc *[12]. As shown in Figure 2D, the up-regulation induced by GabT of genes of the EGR family, *Nr4a1 *and *Arc *was significantly reduced and almost blocked by inhibitors of the ERK1/2 pathway, but not the up-regulation of *Bdnf *and *Homer1a*.

The results described in the present investigation show that when following GabT a potentiation of the evoked electrical activity occurs at medium times (M-LTP). This form of chemically induced LTP is expected to modify the great majority of synapses present in the network and therefore to affect its global properties. When LTP is induced by a local electrical stimulation, only a limited number of synapses are expected to be modified.

As shown in Fig. 2, potentiation of the M-LTP was reduced, but not eliminated by inhibitors of the ERK1/2 pathway, in agreement with the notion that neuronal plasticity is mediated by several distinct pathways likely to be working in unison. These results allowed us to relate changes of electrical properties occurring during neuronal plasticity to specific underlying molecular events.

The present analysis combining MEA and DNA microarrays represents a simple system to study neuronal plasticity [4], but does not allow to identify the cellular origin of detected changes of gene expression. Given the large abundance of pyramidal neurons in hippocampal cultures and acute slices it is likely that detected changes of gene expression occur in these neurons, but it is possible that they occur also in interneurons and in glial cells. In order to resolve this issue it will be necessary to perform single cell gene profiling in the intact hippocampus. Preliminary experiments performed in our laboratory in intact organotypic slices show that treatment with gabazine induces very similar changes of gene expression in dissociated cultures, as those here used and in neuronal slices preserving the original physiological connectivity.

## Methods

### Neuronal culture preparation

Hippocampal neurons dissociated from Wistar rats (P0–P2) were plated on polyorhitine/matrigel pre-coated MEA at a concentration of 8 × 10^5^cells/cm^2 ^and maintained in a neuron medium as previously described [9]. After 48 h, 5 μM cytosine-*β-*D-arabinofuranoside (Ara-C) was added to the culture medium in order to block glial cell proliferation. Neuronal cultures were kept in an incubator providing a controlled level of CO_2 _(5%), temperature (37°C) and moisture (95%).

### Electrical recordings and electrode stimulation

Multi electrode array (MEA) recordings were carried out with a MEA60 system (Multi Channel Systems, Reutlingen, Germany). Stimulations and recordings were carried out after 21–35 days *in vitro*. Synchronous network bursting was induced by 30 min. treatment with GABA_A _receptor antagonists, gabazine (20 μM). In some experiments, blockers of the ERK1/2 pathway (50 μM PD98059 and 20 μM U0126) were used. These drugs were pre-incubated before application of gabazine for 45 min. The voltage pulse was bipolar with a duration of 200 μsec. For a given culture, the same amplitude was used as before, during and after GabT. The pattern of stimulation consisted of a train of 40 bipolar pulses separated by an inter-pulse interval of 4s and applied to a bar of six neighbouring electrodes.

### Data analysis

Acquired data were analyzed using MATLAB (The Mathworks, Inc.) as previously described [10,19]. The *network firing rate *(NFR) is defined as the sum of all electrodes firing rates (i.e. the number of all spikes recorded in the network for each bin). The total number of spikes as a function of the distance d was fitted by the exponential function from which λ was obtained (inset Fig. 1G).

### Quantitative RT-PCR

RNA (250 ng) was reverse transcribed using SuperScript II reverse transcriptase and random hexamer (Invitrogen, Milan, Italy). Real-time PCR was performed using iQ SYBR Green supermix (Biorad, Milan, Italy) and the iQ5 LightCycler. Gene specific primers were designed using Beacon Designer (Premier Biosoft, Palo Alto, CA, USA). The thermal cycling conditions comprised 3 min at 95°C, and 40 cycles of 10 seconds for denaturation at 95°C and 45 sec for annealing and extension at 58°C. The expression level of the target mRNA was normalized to the relative ratio of the expression of *Gapdh *mRNA. The forward primer for *Gapdh *was 5'-CAAGTTCAACGGCACAGTCAAGG-3', the reverse primer was 5'-ACATACTCAGCACCAGCATCACC-3'. Fold changes calculations were made between treated and untreated samples at each time point using the 2^-ΔΔ*CT *^method. The forward primer for *Egr1 *was 5'-AAGGGGAGCCGAGCGAAC-3', the reverse primer was 5'-GAAGAGGTTGGAGGGTTGGTC-3'; forward primer for *Egr2 *was 5'-CTGCCTGACAGCCTCTACCC-3', reverse primer was 5'-ATGCCATCTCCAGCCACTCC-3'; forward primer for *Egr3 *was 5'-ACTCGGTAGCCCATTACACTCAG-3', reverse primer was 5'-GTAGGTCACGGTCTTGTTGCC-3'; forward primer for *Nr4a1 *was 5'-GGTAGTGTGCGAGAAGGATTGC-3', reverse primer was 5'-GGCTGGTTGCTGGTGTTCC-3'; forward primer for *Arc *was 5'-AGACTTCGGCTCCATGACTCAG-3', reverse primer was 5'-GGGACGGTGCTGGTGCTG-3'; forward primer for *Homer1a *was 5'-GTGTCCACAGAAGCCAGAGAGGG-3', reverse primer was 5'-CTTGTAGAGGACCCAGCTTCAGT-3'; forward primer for *Bdnf *was 5'-CGATTAGGTGGCTTCATAGGAGAC-3', reverse primer was 5'-GAAACAGAACGAACAGAAACAGAGG-3'.

## Abbreviations

MEA: multi electrodes array; LTP: long term potentiation; GabT: gabazine treatment; NFR: network firing rate; ERK: extracellular signal regulated; GABA: gamma amino butyric acid; LFP: local field potential

## Competing interests

The authors declare having no financial and no non-financial competing interests.

## Authors' contributions

FB performed and analyzed MEA experiments and prepared the results for publication. SP performed and analyzed the molecular biology experiments, performed part of the MEA experiments, interpreted and prepared the results for publication. MER participated in the design and interpretation of the experiments and prepared the results for publication. CA carried out statistical analysis of the microarrays data. VT supervised the project, participated in its design, coordination and drafting of the manuscript. FB, SP, MER and VT wrote the manuscript. All authors read and approved the final version of the manuscript.
